# The role of HIV-1 Gag and genomic RNA interactions in virion assembly

**DOI:** 10.3389/fmicb.2025.1642090

**Published:** 2025-08-05

**Authors:** Takaaki Koma, Bao Quoc Le, Khanh Quoc Tran, Naoya Doi, Tomoyuki Kondo, Kei Miyakawa, Akio Adachi, Masako Nomaguchi

**Affiliations:** ^1^Department of Microbiology, Graduate School of Medicine, Tokushima University, Tokushima, Japan; ^2^Division of Interdisciplinary Researches for Medicine and Photonics, Institute of Post–LED Photonics, Tokushima University, Tokushima, Japan; ^3^AIDS Research Center, National Institute of Infectious Diseases, Japan Institute for Health Security, Tokyo, Japan

**Keywords:** HIV-1, virion assembly, Gag, RNA, membrane, Gag oligomerization, Gag multimerization

## Abstract

The virion assembly represents a critical aspect of producing infectious progenies required for HIV-1 replication. Each step in that process, such as Gag-membrane binding, Gag-genomic RNA binding/packaging, Gag multimerization, and viral particle budding, has been extensively analyzed in a stepwise and specific manner. While Gag proteins are the primary drivers of HIV-1 virion assembly, the interactions between Gag and RNA play a significant role in regulating the process. This article provides an updated overview and perspective on HIV-1 virion assembly, with a particular focus on the role of Gag-RNA interactions.

## 1 Introduction

HIV-1 has been spreading all over the world. Around 39.9 million people were living with HIV, and around 1.3 million people became newly infected with HIV in 2023 (Global HIV & AIDS statistics – Fact sheet 2024 UNAIDS).^1^ While no HIV vaccine has been established yet, effective antiretroviral therapy (ART) can control viral replication in infected patients and prevent the development of AIDS. However, ART also has issues including virologic failure, immunologic non-responder, and HIV drug resistance (Lehman et al., 2023; SeyedAlinaghi et al., 2023; Esteban-Cantos et al., 2024; Guedes et al., 2025; Nuwagaba et al., 2025). To effectively tackle them, continued efforts are required to develop novel antiviral drugs and therapies, grounded in a deeper understanding of HIV-1 replication, mutation, and adaptation.

HIV-1 replication is divided into two phases, the early replication phase and the late replication phase (Freed, 2004). The early replication phase is initiated by viral entry into host cells via the binding to receptor CD4 and coreceptor CCR5 and/or CXCR4. Then, HIV-1 virion-associated reverse transcriptase synthesizes double-stranded DNA from viral RNA genome and the newly synthesized viral DNA is integrated into host cell chromosome by catalytic action of integrase contained in the preintegration complex (see reviews, Craigie and Bushman, 2012; Hu and Hughes, 2012; Arribas et al., 2024; Dwivedi et al., 2024). The late replication phase includes viral gene expression from integrated viral DNA and virion assembly (see reviews, Bieniasz, 2009; Karn and Stoltzfus, 2012; Sundquist and Kräusslich, 2012; Freed, 2015; Sumner and Ono, 2022; Sumner and Ono, 2024). The HIV-1 gene expression is a tightly regulated process that includes transcription, alternative splicing, mRNA export, and translation. The full-length viral RNA serves not only as viral genome RNA (gRNA) incorporated into progeny virions but also as an mRNA coding viral structure proteins Gag and Gag-Pol. Recent studies showed that there are two distinct 5′ isoforms of full-length viral RNAs: the one starts with 1G, which functions as the gRNA encapsidated, and the other one carrying 3G serves as an mRNA (Masuda et al., 2015; Kharytonchyk et al., 2016). Although HIV-1 virion assembly is initiated by the binding of Gag and gRNA (Bieniasz and Telesnitsky, 2018; Duchon and Hu, 2024), the mechanisms underlying the virion assembly process have not yet been fully elucidated. In this article, we summarize the HIV-1 assembly process with a focus on Gag and RNA interactions, developing perspectives on the relevant research based on the summary.

## 2 Overview of HIV-1 virion assembly

HIV-1 virion assembly proceeds with several steps: Gag and gRNA interactions, Gag multimerization including a small amount of Gag-Pol on the plasma membrane (PM), and immature Gag lattice formation. Eventually, HIV-1 immature particles harbor the Gag lattice containing two copies of gRNA and envelope proteins. After budding from cells, immature particles turn into mature virions through cleavage of Gag mediated by Gag-Pol coding protease (see reviews, Bieniasz, 2009; Sundquist and Kräusslich, 2012; Freed, 2015).

**FIGURE 1:**
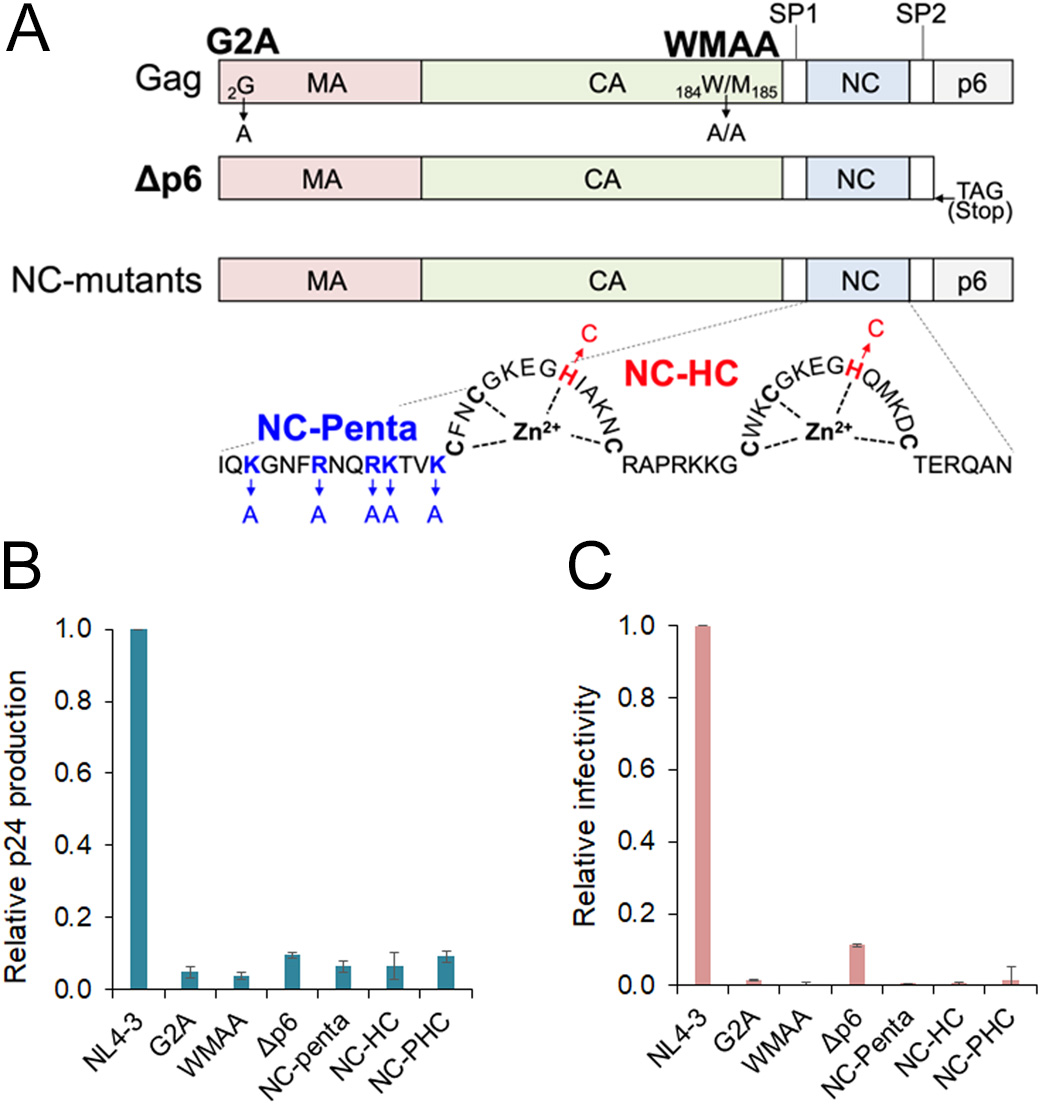
Effect of Gag mutations on HIV-1 virion production and viral infectivity. **(A)** Gag mutants used in this study. Gag organization is presented with colored domains. Bold letters show Gag mutant names. G2A and WMAA mutants contain the indicated mutations. A deletion mutant Δp6 has a stop codon at the end of SP2 domain. Two NC mutants, NC-Penta and NC-HC, harbor the indicated K/R to A mutations (blue) and H to C mutations (red), respectively. NC-PHC, which carries all mutations in the NC-Penta and NC-HC, was also generated in this study. **(B)** Virion production. Because of the highly sensitive nature to transfection, HEK293T cells were used here for easy comparison of the differences between WT and the Gag mutants. The cells were transfected with the indicated proviral clones, and on day 1 post-transfection, the supernatants were collected for analysis. Virus amounts were measured by the HIV-1 p24 antigen ELISA kit (ZeptoMetrix Corporation). Relative virion production of Gag mutants to that of WT NL4-3 from at least three independent experiments is presented. **(C)** Viral infectivity. Infectivity assays using TZM-bl cells were performed as previously described (Nomaguchi et al., 2016). Viral infectivity is presented as relative values to that of WT NL4-3 (*n* = 3).

HIV-1 Gag protein is the main driver of virion assembly. Gag consists of matrix (MA), capsid (CA), SP1, nucleocapsid (NC), SP2, and p6, arranged from the N-terminus to the C-terminus (Figure 1A) (see reviews, Bieniasz, 2009; Sundquist and Kräusslich, 2012; Freed, 2015). Gag-MA primarily functions in PM binding via myristoylation at the N-terminal glycine and a highly charged patch of highly basic region (HBR). Gag-CA exhibits the ability of adjacent CA-CA interactions and is a driving force of immature lattice formation and subsequent virus assembly. Gag-CA is divided into the N-terminal domain (NTD) and C-terminal domain (CTD). Gag-CA-CTD forms a six-helix bundle with its downstream SP1. Gag-NC contains two zinc finger motifs and flanking basic residues and mediates RNA binding. The RNA binding ability of Gag-NC contributes to immature lattice formation and the selective packaging of two copies of gRNA into viral particles. Gag-p6 has PTAP and YPXL-type binding motifs and plays a role in fission between virus particles and cellular membranes by utilizing host cellular ESCRT machinery.

As previously reported by many researchers, we ourselves generated several Gag mutants to confirm and validate the importance of Gag residues (Figure 1A): G2A, disruption of myristoylation in Gag-MA and subsequent PM targeting (Göttlinger et al., 1989; Bryant and Ratner, 1990; Freed et al., 1994); WMAA, defect in Gag dimerization by mutations (W184A and M185A) in Gag-CA-CTD domain (Gamble et al., 1997; von Schwedler et al., 2003); Δp6, deficiency in interactions between Gag-p6 and host TSG101/ESCRT machinery to facilitate the virus budding (Demirov et al., 2002; Pornillos et al., 2002); NC-HC (the zinc finger motifs mutant) and NC-Penta (the basic region mutant), loss of RNA binding ability, especially via specific binding to the psi element on the gRNA and electrostatic non-specific binding to nucleic acids, respectively (Dannull et al., 1994; Schmalzbauer et al., 1996). NC-PHC is a double mutant carrying all mutations in NC-HC and NC-Penta. As shown in Figures 1B, C, virion production and infectivity in all mutants tested were drastically reduced compared to those in wild type (WT). These results, in line with the previous reports, verify the critical roles of each Gag domain in HIV-1 assembly.

## 3 Initiation of HIV-1 virion assembly process: Gag-gRNA association

HIV-1 virions contain two copies of gRNA, which are essential for the formation of infectious progenies. The packaging of dimerized gRNA into virions results from the binding to gRNA via Gag-NC, and this Gag-gRNA interaction is a starting point of HIV-1 assembly (Bieniasz and Telesnitsky, 2018; Duchon and Hu, 2024). Despite many reports published, the initial phase of HIV-1 virion assembly, especially when, where, and how gRNA dimerization and Gag-gRNA interactions occur, is still one of the mysteries.

In HIV-1 infected cells, Gag needs to select gRNA from abundant cellular RNAs, maintaining the interaction with dimerized gRNA on the PM for efficient gRNA packaging and infectious virion production. The Gag binding to gRNA is mediated by a high affinity interaction between two zinc-finger motifs in Gag-NC and the psi element on gRNA for the selective packaging of gRNA (Comas-Garcia et al., 2016; Rein, 2019; Bieniasz and Telesnitsky, 2018; Duchon and Hu, 2024). Since Gag-NC alone is not enough but Gag-CA-NC is required for the binding to psi element (Lei et al., 2023), Gag multimerization by CA-CA interactions has been suggested to affect the ability of Gag binding specific to gRNA *in vitro* and cell-based assays (Roldan et al., 2004; Lei et al., 2023). The sequence surrounding the psi element is also important for the selective packaging of gRNA. It has been shown that the upstream sequence from 5′ UTR to the *gag* gene facilitates gRNA packaging (Richardson et al., 1993; Berkowitz et al., 1995; Kaye et al., 1995; Chamanian et al., 2013; Liu et al., 2017).

Gag proteins present in the cytoplasm as monomers and/or low-order multimers are transported to the PM mainly by diffusion (Kutluay and Bieniasz, 2010; Fogarty et al., 2014; Chen et al., 2014; Hendrix et al., 2015). Even in the absence of Gag, gRNA can reach the PM, whereas HIV-1 RNA can stay longer at the PM in the presence of Gag. Gag-NC-deleted or non-myristoylated Gag mutants, and also gRNA packaging signal mutants cannot keep HIV-1 RNA at the PM (Jouvenet et al., 2009; Chen et al., 2014; Sardo et al., 2015), suggesting that Gag binding is necessary for retaining gRNA at the PM. An early imaging study suggested that dimerized gRNA arrived at the PM as complexes with a small amount of Gag (Jouvenet et al., 2009). Imaging studies also revealed that the gRNA dimerization occurs in the cytosol (Ferrer et al., 2016) and that Gag oligomers and RNA-interacting Gag oligomers are present in the cytosol (Jouvenet et al., 2009). On the other hand, it has been shown by the live-cell imaging approach that the site of gRNA dimerization can be at the PM (Chen et al., 2016) and by the chemical crosslinking technique that Gag binding to the psi element can occur at the PM or in the cytosol (Lei et al., 2023). Gag appears to facilitate gRNA dimerization in the cytoplasm (Hendrix et al., 2015) and seems to stabilize dimerized gRNA at the PM (Chen et al., 2016).

## 4 Gag binding to the PM

Gag binding to the PM mediated by Gag-MA is a step that proceeds HIV-1 virion assembly. Myristoylation at the N-terminus of Gag-MA is essential for binding to the PM and HIV-1 virion production (Göttlinger et al., 1989; Bryant and Ratner, 1990; Freed et al., 1994). It has been suggested that while the myristate moiety at the N-terminus of Gag-MA is sequestered to prevent Gag and membrane interactions, myristate exposure was enhanced by promoting Gag-CA self-assembly (Sandefur et al., 1998; Lindwasser and Resh, 2001; Perez-Caballero et al., 2004; Tang et al., 2004). Gag oligomerization seems to induce myristate exposure and stabilize membrane binding of Gag.

In addition to the myristate moiety, a positively charged patch HBR in Gag-MA also facilitates PM binding of Gag. Gag-MA-HBR binds to phosphatidylinositol-(4,5)-bisphosphate [PI(4,5)P2], which is a highly negatively charged lipid localized predominantly at the inner leaflet of the PM (Saad et al., 2006; Shkriabai et al., 2006; Chukkapalli et al., 2008; Mercredi et al., 2016). It has been suggested that PI(4,5)P2 is involved in HIV-1 virion assembly and production by: (1) determining cellular Gag localization, (2) contributing to Gag targeting to PM, and (3) contributing to stable associations between Gag and PM via its interactions with Gag-MA (Ono et al., 2004; Jouvenet et al., 2006; Saad et al., 2006; Shkriabai et al., 2006; Chukkapalli et al., 2008; Mercredi et al., 2016; Mücksch et al., 2017).

Gag-MA has the ability to interact with nucleic acid through its HBR (Ott et al., 2005; Shkriabai et al., 2006; Alfadhli et al., 2009; Chukkapalli et al., 2010). Gag-MA has been described to bind to viral RNAs *in vitro* and cell-based assays, and to make a Gag compact form by its simultaneous binding to the viral RNA together with Gag-NC (Shkriabai et al., 2006; Datta et al., 2007; Zeiger et al., 2024). RNA crosslinking-immunoprecipitation study reported that Gag-MA binding to viral RNA was not observed and that major RNA species that Gag-MA binds in cells were tRNA, especially tRNA (Gly, Lys, and Val) (Kutluay et al., 2014). Further studies are required to elucidate the involvement of the Gag compact form and the RNA species bound by Gag-MA in the HIV-1 virion assembly. Meanwhile, Gag-MA-HBR has been shown to exhibit higher affinity for PI(4,5)P2 than nucleic acids (Chukkapalli et al., 2008; Alfadhli et al., 2009; Chukkapalli et al., 2010; Chukkapalli et al., 2013). Thus, the binding of Gag-MA to tRNAs appears to prevent Gag binding to intracellular membranes containing not much PI(4,5)P2 and seems to enhance specific binding to the PM enriched with PI(4,5)P2 (Bieniasz and Telesnitsky, 2018; Sumner and Ono, 2022; Duchon and Hu, 2024).

As described above, while Gag-Gag interactions contribute to myristate exposure and PM targeting (Sandefur et al., 1998; Lindwasser and Resh, 2001; Perez-Caballero et al., 2004; Tang et al., 2004), it seems that Gag with the low concentration localizes in the cytosol, as increasing concentration of Gag reaches the PM and stably binds to the PM through Gag oligomerization (Perez-Caballero et al., 2004; Fogarty et al., 2014). This implies that Gag-Gag interactions themselves can enhance the PM binding.

## 5 Gag multimerization on the PM

After stable binding of Gag with dimeric gRNA to the PM, Gag multimerization proceeds toward immature viral particle formation on the PM. The main driver of Gag multimerization is a self-assembly via Gag CA-CA interactions. As described above, this Gag multimerization promotes stable membrane binding.

Gag-NC possesses basic R and K containing regions surrounding zinc-finger domains (Figure 1A). The basic regions of Gag-NC can electrostatically interact with positively charged nucleic acids (Rein, 2019; Bieniasz and Telesnitsky, 2018). Mutations of Gag-NC basic regions have been reported to reduce virion production (Dawson and Yu, 1998; Cimarelli and Luban, 2000). While in *in vitro* assays, the presence of nucleic acids promotes efficient Gag assembly, the nucleic acid binding ability of Gag-NC facilitates Gag multimerization on the PM by utilizing RNAs as scaffolds (Campbell and Rein, 1999; Pak et al., 2017; Yang et al., 2018). Although HIV-1 can produce virus-like particles by Gag assembly using any RNA in the absence of gRNA, under the regulated condition of Gag expression at a similar level as that in infected cells, gRNA has been shown to promote viral assembly and virion production (Dilley et al., 2017; Ying et al., 2024). Thus, specific interactions between Gag and gRNA are important for Gag targeting to PM and subsequent stable association, being crucial for retaining dimeric gRNA and its incorporation into virions. Furthermore, since HIV-1 RNA possesses Gag binding sites other than the psi element, many Gag proteins can bind to dimeric gRNA scaffolding and thereby facilitate Gag-Gag interactions (Dilley et al., 2017). Taken together, Gag and gRNA interactions are crucial for HIV-1 virion assembly.

## 6 Discussion

Interactions of HIV-1 Gag and dimeric gRNA seed immature Gag lattice formation and nucleate Gag assembly on the PM. Furthermore, gRNA serves as a scaffold for Gag multimerization at the PM (Nikolaitchik et al., 2013; Dilley et al., 2017; Pak et al., 2017; Yang et al., 2018; Ying et al., 2024). Although Gag and dimerized gRNA interactions are well known to play a pivotal role in HIV-1 virion assembly, further studies are necessary to elucidate when, where, and how they first encounter in cells.

**FIGURE 2:**
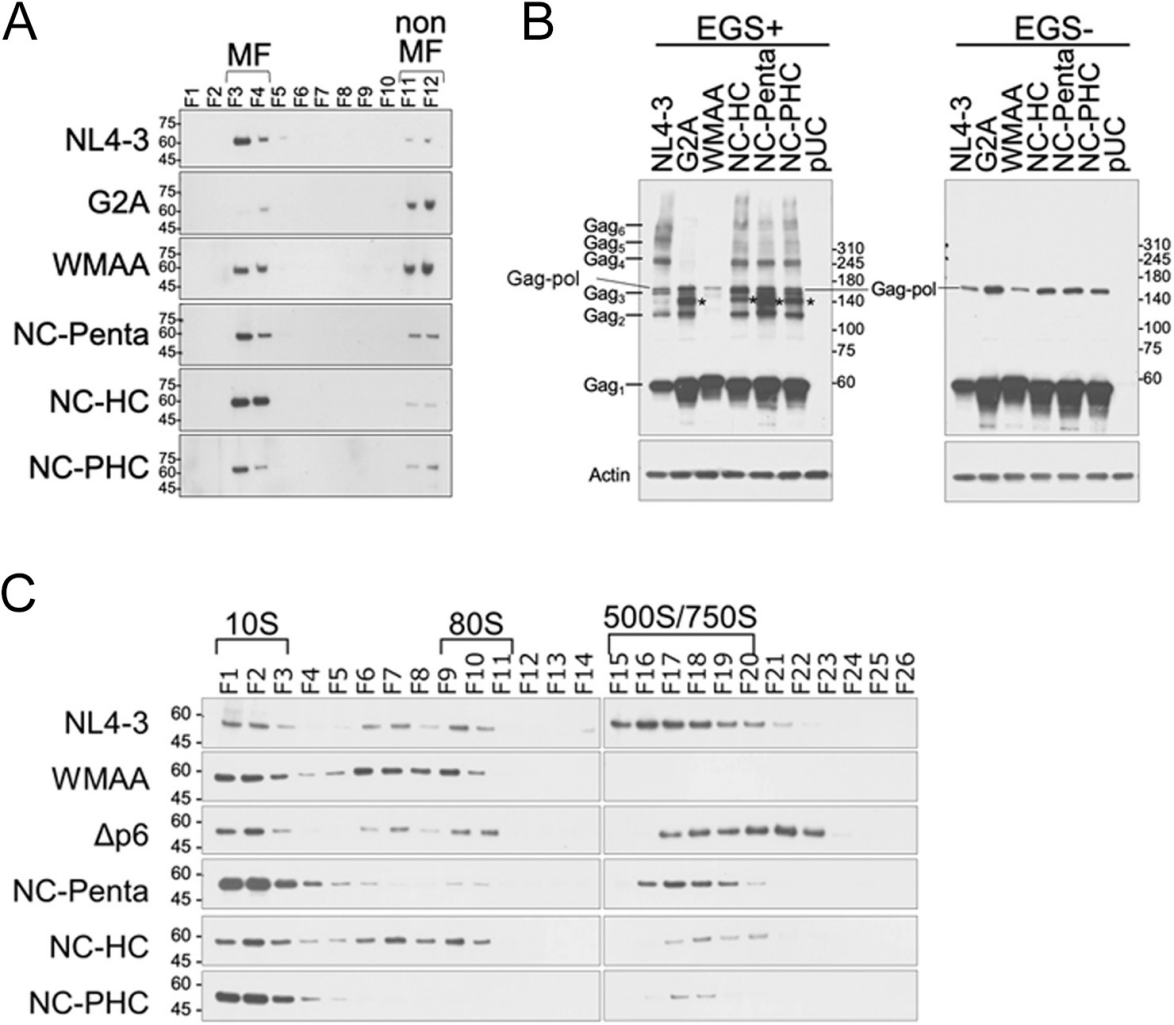
Effect of various Gag mutations on the ability of membrane binding and multimerization. Since HeLa cells are known to be better suited than HEK293T cells for subtle analytical quantitative studies, HeLa cells were used in the following experiments. **(A)** Membrane flotation assay. The assays were performed using the indicated clones as previously described (Tanaka et al., 2015; Koma et al., 2019). Representative data from three independent experiments are shown. MF, membrane fractions. **(B)** Chemical crosslinking assay. EGS [ethylene glycol bis (succinimidyl succinate)] used for the assays is a homobifunctional and membrane-permeable crosslinker. The assays were performed using the indicated clones as previously described (Kutluay and Bieniasz, 2010). Representative data from three independent experiments are shown. **(C)** Velocity sedimentation assays. The assays were performed using the indicated clones as previously described (Koma et al., 2019). Representative immunoblotting data from at least three independent experiments are shown. All assays were carried out by transfection of ΔProteaseΔEnvelope proviral clones into HeLa cells using Lipofectamine 2000 (Thermo Fisher Scientific).

We examined the effect of Gag-gRNA interactions on Gag membrane binding and Gag oligomerization/multimerization using Gag mutants (G2A, WMAA, NC-Penta, NC-HC, NC-PHC, and/or Δp6) (Figure 1A). Membrane flotation assays enable us to separate membrane and non-membrane fractions (Tanaka et al., 2015; Koma et al., 2019). Gag expression levels in cells used for the membrane flotation assays were similar among samples tested (Supplementary Figure 1). The assays showed that G2A and WMAA exhibited higher amounts of Gag in non-membrane fractions compared to WT, whereas Gag distributions of three NC mutants are quite similar to those of WT (Figure 2A). WMAA mutations have been shown to disrupt stable Gag-PM binding and significantly decrease virion production (Ono et al., 2005; Joshi et al., 2006; Klein et al., 2011; Robinson et al., 2014). In Gag-NC zinc-finger mutants, the accumulation of Gag at the PM was also observed (Grigorov et al., 2007). Our data from membrane flotation assays are thus consistent with these previous results.

In our chemically crosslinking assays (Figure 2B) to assess Gag dimer to hexamer formation, non-membrane bound G2A and WMAA mutants showed inhibited Gag oligomerization as previously reported (Kutluay and Bieniasz, 2010). Especially for the mutant WMAA, even Gag dimers were not detected. In contrast to G2A and WMAA mutants, three Gag-NC mutants tested exhibited Gag band patterns similar to those of WT. Since Gag with NC mutations accumulated at the PM (Figure 2A), these results for Gag-NC mutants coincide with Gag membrane targeting and stable association with the PM by Gag-Gag interactions. Cell-based nanoBRET assays also supported the presence of Gag-Gag interactions for NC-HC but not for G2A or WMAA (Supplementary Figure 2). We noticed that several kinds of bands were detected in Gag mutants but not in WT (* in Figure 2B), although we did not analyze the origin of the bands.

To further monitor the intracellular assembly process of mutant Gag proteins, we performed the velocity sedimentation assay using sucrose gradient ultracentrifugation (Klein et al., 2011; Robinson et al., 2014; Tanaka et al., 2015; Barajas et al., 2018; Koma et al., 2019). With this assay, Gag proteins are fractionated by their sedimentation values (10S, 80S, 150S, 500S, and 750S). When Gag mutations are detrimental to a step in the assembly process, Gag proteins are not readily detected in the fractions corresponding to the step. As shown in Figure 2C, Supplementary Figure 3, as virus assembly proceeds (WT NL4-3), the bands of Gag were readily detectable in 500S/750S fractions, which represent Gag multimerization at the PM. It has been shown that G2A exhibits accumulation of the 10S–80S complexes, but not of other fractions (Robinson et al., 2014; Tanaka et al., 2015). A control mutant WMAA with the defect in Gag dimerization and stable membrane binding exhibited no detectable amounts of Gag in 500S/750S fractions, whereas another control mutant Δp6 with the deficiency in virus particle release could form the 500S/750S Gag complex (Figure 2C). As expected, Gag proteins of Gag-NC mutants did not accumulate in 500S/750S fractions unlike those of WT and Δp6. These results suggested that Gag-NC mutants have the ability to reach the PM and to form hexameric Gag by Gag-CA interactions like WT, which is the process independent of their RNA binding ability (Figures 2A, B). However, these mutants are incapable of further multimerized Gag formation at the PM that requires Gag-NC and RNA interactions, as observed by their lower intensity of 500S/750S Gag fractions (Figure 2C). Thus, the ability of Gag to bind RNA would be associated with assembly Gag lattice/virion production by facilitating nucleation of Gag assembly and/or efficient Gag multimerization at the PM.

It has been proposed that the 80S Gag complex observed in the velocity sedimentation assay can be an assembly intermediate (Barajas et al., 2018). However, later sucrose gradient analysis suggested that the 80S complex may not be a virion assembly intermediate because it could be ribosome complexes bound to monomeric or dimeric Gag proteins (Deng et al., 2021). While Gag-NC mutants, NC-Penta and NC-PHC, exhibited similar Gag expression levels in cells to WT (Supplementary Figure 3), Gag protein levels in 80S fractions for the two NC mutants decreased compared to WT (Figure 2C). This might be due to the disruption of stable Gag and gRNA/cellular RNA binding in these Gag-NC mutants. Thus, the Gag-containing complex in 80S fractions might be involved in HIV-1 virion assembly. Although the 80S complex could be RNA granules containing Gag, gRNA, and RNA binding proteins (Barajas et al., 2018), its exact nature remains to be elucidated. Meanwhile, the role of liquid-liquid phase separation in Gag assembly has been reported (Monette et al., 2020; Zhang et al., 2023). It remains an intriguing issue as to where and how the Gag–RNA complex is assembled: the potential involvement of RNA granules and other biomolecular condensates.

## 7 Concluding remarks

Biochemical approaches, the *in vitro* assembly/*in vivo* crosslinking methods and the imaging analyses that visualize Gag/gRNA, have contributed significantly to clarify the mechanism for each step involved in the HIV-1 virion assembly. However, variations in the analytical methods may occasionally lead to different interpretations. Host proteins such as IP6 that play a role in HIV-1 virion assembly have also been identified recently (Lerner et al., 2022; Sumner and Ono, 2022; Duchon and Hu, 2024; Lacouture et al., 2024; Sumner and Ono, 2024). Considering that gRNA promotes HIV-1 assembly and virion production under the condition of Gag expression levels regulated as in infected cells (Dilley et al., 2017), the assembly process needs to be analyzed using HIV-1 infected cells in more detail, including the involvement of Gag-RNA complexes and host factors. A deeper understanding of the assembly process would contribute to the establishment of novel techniques and antivirals to control HIV-1 replication.

## Data Availability

The original contributions presented in this study are included in this article/Supplementary material, further inquiries can be directed to the corresponding authors.

## Footnotes

1
https://www.unaids.org/en/resources/fact-sheet
