# Supplementary material for: The role of HIV-1 Gag and genomic RNA interactions in virion assembly
Source: Front Microbiol. 2025 Aug 5;16:1642090. doi: 10.3389/fmicb.2025.1642090 (PMC12392317; doi:10.3389/fmicb.2025.1642090)
Supplement: Supplementary file 1 [file Data_Sheet_1.pdf]

*Supplementary Material*

# The role of HIV-1 Gag and genomic RNA interactions in virion assembly

**Takaaki Koma[1,2], Bao Quoc Le[1], Khanh Quoc Tran[1], Naoya Doi[1], Tomoyuki Kondo[1], Kei Miyakawa[3], Akio Adachi[1*], Masako Nomaguchi[1,2*]**

[1]Department of Microbiology, Graduate School of Medicine, Tokushima University, Tokushima, Japan

[2]Division of Interdisciplinary Researches for Medicine and Photonics, Institute of Post−LED Photonics, Tokushima University, Tokushima, Japan,

[3]AIDS Research Center, National Institute of Infectious Diseases, Japan Institute for Health Security, Tokyo, Japan

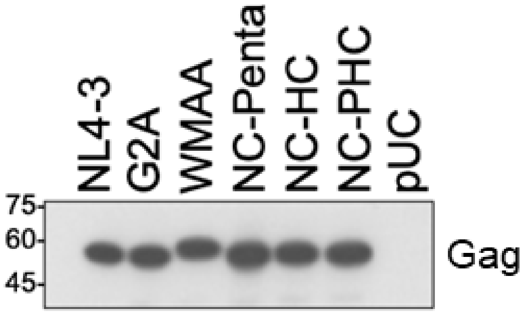


**Supplementary Figure 1.** Gag expression levels in input samples used for membrane flotation analysis. Cell lysates were prepared as described in Figure 2A and subjected to the Western blotting analysis using an anti-p24 antibody (183-H12-5C) (NIH AIDS Reagent Program). Representative data from at least three independent experiments are shown.

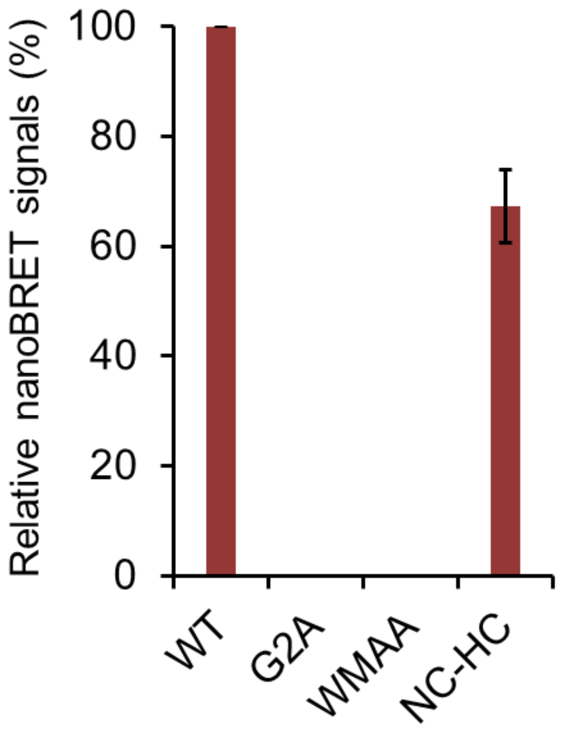


**Supplementary Figure 2.** Analysis of Gag-Gag interactions by nanoBRET assays. These assays were performed using HEK293T cells as previously described (Miyakawa et al., 2017; Koma et al., 2019). Relative values of NanoBRET signals to that of WT are presented (*n* = 6, triplicate samples in two independently performed experiments).

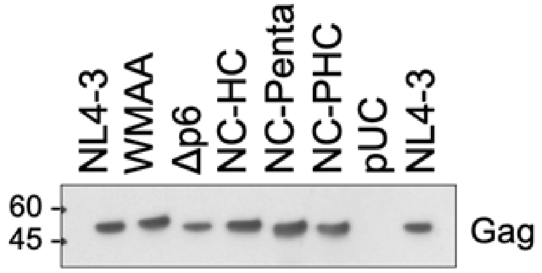


**Supplementary Figure 3.** Gag expression levels in input samples used for velocity sedimentation assays. Cell lysates were prepared as described in Figure 2C and subjected to the Western blotting analysis using an anti-anti-p24 antibody (183-H12-5C) (NIH AIDS Reagent Program). Representative data from at least three independent experiments are shown.

**References**

Miyakawa K, Nishi M, Matsunaga S, Okayama A, Anraku M, Kudoh A,et al. (2017). The tumour suppressor APC promotes HIV-1 assembly via interaction with Gag precursor protein. *Nat Commun* 8:14259. doi: 10.1038/ncomms14259

Koma T, Kotani O, Miyakawa K, Ryo A, Yokoyama M, Doi N, et al., (2019). Allosteric regulation of HIV-1 capsid structure for Gag assembly, virion production, and viral infectivity by a disordered interdomain linker. *J Virol* 93:e00381-19. doi: 10.1128/JVI.00381-19.
